# A Rare Case of Synchronous Multiple Primary Malignancies of the Pancreas, Skin, and Thyroid

**DOI:** 10.1155/crom/9577921

**Published:** 2025-12-30

**Authors:** Michael Barsoum, Crystal Antoine Pepeljugoski

**Affiliations:** ^1^ Department of Internal Medicine, Northwell Health Northern Westchester Hospital, Mount Kisco, New York, USA; ^2^ Department of Hematology/Oncology, Northwell Health Northern Westchester Hospital, Mount Kisco, New York, USA

## Abstract

Multiple primary malignancies (MPMs) are defined as the occurrence of two or more distinct malignant neoplasms in a single individual, arising either simultaneously or successively. With advancements in targeted therapies and improvements in overall survival, the observed incidence of MPMs has increased, likely reflecting both improvements in oncologic treatments and enhanced surveillance. We report the case of a 63‐year‐old man with synchronous pancreatic neuroendocrine tumor, anal melanoma, and papillary thyroid carcinoma. His prior oncologic history is notable for prostate adenocarcinoma treated with prostatectomy and renal cell carcinoma managed with right partial nephrectomy. Over a 1‐year period, he underwent surgical resection of the neuroendocrine tumor, anal melanoma, and papillary thyroid carcinoma. He is currently receiving pembrolizumab for management of anal melanoma. Germline testing using Ambry′s CustomNext Cancer panel was negative across 33 genes associated with hereditary cancer predisposition syndromes. Synchronous MPMs pose substantial challenges for clinical management, including complexities in treatment prioritization, sequencing, and integration of multimodal therapies. These challenges are compounded by limited evidence to guide concurrent or sequential multicancer regimens, with implications for efficacy, toxicity, and long‐term outcomes. This case underscores the need for multidisciplinary coordination, individualized risk–benefit assessment, and prospective data to inform evidence‐based strategies in the care of patients with MPMs.

## 1. Introduction

Multiple primary malignancies (MPMs) refer to the presence of two or more malignant tumors in an individual, either simultaneously or successively. MPMs are classified into three categories: Group I, multiple primary tumors within the same organ; Group II, MPMs originating in different organs or tissues; and Group III, tumors from different organs and tissues, also encompassing those of Group I. Further classification divides MPMs into synchronous, malignancies observed at the same time or within 6 months, and metachronous, those observed after a 6‐month interval [1–3]. This report presents a patient with a history of prostate cancer and renal cell carcinoma who subsequently presented with synchronous pancreatic neuroendocrine tumor, anal melanoma, and papillary thyroid carcinoma.

## 2. Case

A 63‐year‐old male with a history of prostate cancer status post prostatectomy in 2012, renal cell carcinoma status post right partial nephrectomy in 2021, and strong family history of multiple malignancies presented to the oncology clinic for evaluation and treatment of a pancreatic neuroendocrine tumor. The patient underwent yearly surveillance MRI for his renal cell carcinoma, which revealed a posterior gastric wall mass and a subcentimeter cystic focus in the pancreatic tail without enhancement. Endoscopic ultrasound showed a subepithelial lesion in the antrum. Biopsy of this lesion revealed small fragments of a spindle cell tumor with a small lymphoid aggregate. A 23‐mm pancreatic tail lesion was also biopsied, showing a non–small cell neuroendocrine neoplasm.

One month later, the patient underwent a pancreatectomy and splenectomy. Pathology of the pancreatic tumor revealed a 3 cm, T2N1, well‐differentiated, G2 neuroendocrine tumor with two out of seven lymph nodes positive and a Ki‐67 labeling index of 3%–20%. A gastric resection performed concurrently revealed a schwannoma. Laboratory results were significant for an elevated C‐peptide of 6.7 ng/mL and an elevated 5‐HIAA of 6.5 mg/24 h. Postpancreatectomy surveillance with a ^68^Ga‐DOTATATE PET scan showed uptake in the parafalcine region of the right frontal lobe, concerning for meningioma, but no evidence of recurrent neuroendocrine tumor.

Concurrently, the patient was evaluated by surgery for removal of a hyperpigmented hemorrhoid with an atypical appearance extending beyond the anal verge into the beginning of the anal canal. Pathology of the anal mass revealed a pT3N0M0, Stage IIB, BRAF‐negative anal melanoma. A subsequent PET scan performed to assess for residual melanoma showed a sclerotic bony lesion at L5 with uptake, mild uptake within several right inguinal lymph nodes, and intense activity in a large thyroid nodule.

Oncology recommended inguinal lymph node biopsy and possible reexcision of the anal melanoma for staging. The patient was also referred to endocrinology for evaluation of the thyroid nodule. Inguinal lymph node biopsies were negative for melanoma. Given his diagnosis of Stage IIB melanoma, he was subsequently started on adjuvant Keytruda with the plan to complete for 1 year. He also underwent a right partial thyroidectomy, with pathology revealing a 1.3 cm pT1bNx papillary carcinoma, for which he is undergoing active surveillance.

Due to his history of multiple primary cancers, he was seen by a cancer genetics specialist. Ambry′s CustomNext Cancer panel, analyzing 33 genes (ATM, BAP1, BRCA1, BRCA2, CDKN2A, CHEK2, EPCAM, FH, FLCN, HOXB13, KIT, LZTR1, MAX, MEN1, MET, MLH1, MSH2, MSH6, NF1, NF2, PALB2, PDGFRA, PMS2, PTEN, SDHA, SDHB, SDHC, SDHD, SMARCB1, STK11, TSC1, TSC2, and VHL) for hereditary cancer syndromes, was negative for clinically significant variants.

## 3. Discussion

Reported incidence estimates for MPMs vary widely across studies, ranging from approximately 2% to 17%. The frequency declines further for patients with triple or quadruple primary malignancies, estimated at 0.5% and 0.3%, respectively [4, 5]. The prevalence of MPMs has increased over time, likely reflecting improvements in cancer detection, therapeutic efficacy, and overall patient longevity.

The etiologic basis of MPMs remains incompletely understood and is plausibly multifactorial, involving genetic predisposition, environmental exposures, and lifestyle‐associated risk factors [2, 4]. The patient described herein had substantial tobacco exposure (one pack per day for 40 years) and a notable family history of malignancy, both of which may have contributed to elevated cancer risk. Given his family history, germline testing with a 33‐gene hereditary cancer panel revealed no pathogenic or likely pathogenic variants consistent with a known hereditary cancer syndrome.

In a cohort study by Borja et al., comprising 2894 individuals with three or more primary malignancies, approximately 35% were found to harbor a recognized hereditary cancer syndrome [6]. While a significant proportion of MPMs can be attributed to established germline predisposition, many cases do not map to currently characterized syndromes. Moreover, genetic testing may identify variants of uncertain significance (VUS), the clinical relevance of which is indeterminate at the time of detection. Notably, a study by Zawar et al. indicated that up to 91% of VUS are ultimately deemed benign, whereas approximately 9% are reclassified as pathogenic [7]. In the present case, no hereditary cancer syndrome was identified; however, the absence of detected pathogenic variants does not preclude an underlying genetic predisposition, given evolving knowledge of cancer‐associated genes and the potential for future reclassification as genomic databases expand.

Although not applicable to this patient′s treatment course, synchronous MPMs present substantial clinical complexity, including challenges in prioritizing and sequencing therapy, as well as uncertainties regarding cumulative efficacy and toxicity. The concurrent administration of multiple cancer‐directed regimens remains insufficiently studied, with limited empirical data to guide evidence‐based decision‐making in patients requiring simultaneous treatment for distinct malignancies.

Continued advancements in diagnostic methodologies and the proliferation of molecularly targeted therapies will likely contribute to further increases in the recognition and reported incidence of MPMs. Further research is needed to elucidate optimal management strategies, refine risk stratification, and clarify the genetic architecture underlying MPMs.

## 4. Conclusion

This case highlights an uncommon presentation of synchronous MPMs involving the pancreas, skin, and thyroid. While a subset of MPMs is attributable to recognized hereditary cancer syndromes, a substantial proportion lacks an identifiable germline etiology, potentially due to undetected pathogenic variants or the presence of VUS. Although this patient achieved disease control with sequential, site‐specific therapies, many individuals with synchronous MPMs require concurrent, multimodal treatment approaches. Given the rising recognition of MPMs driven by improved diagnostics and prolonged survival, prospective studies are needed to evaluate the efficacy, safety, and optimal sequencing of concurrent regimens in patients with MPMs.

## Conflicts of Interest

The authors declare no conflicts of interest.

## Funding

No funding was received for this manuscript.

## Data Availability

The data that support the findings of this study are available upon request from the corresponding author. The data are not publicly available due to privacy or ethical restrictions.

## References

[1] Vogt A. , Schmid S. , Heinimann K. , Frick H. , Herrmann C. , Cerny T. , and Omlin A. , Multiple Primary Tumours: Challenges and Approaches, a Review, ESMO Open. (2017) 2, no. 2, e000172, 10.1136/esmoopen-2017-000172, 2-s2.0-85041051593, 28761745. 28761745 PMC5519797

[2] Luciani A. and Balducci L. , Multiple Primary Malignancies, Seminars in Oncology. (2004) 31, no. 2, 264–273, 10.1053/j.seminoncol.2003.12.035, 2-s2.0-1842782760. 15112155

[3] Jena A. , Patnayak R. , Lakshmi A. Y. , Manilal B. , and Reddy M. K. , Multiple Primary Cancers: An Enigma, South Asian Journal of Cancer. (2016) 5, no. 1, 29–32, 10.4103/2278-330X.179698. 27169120 PMC4845605

[4] Zhang B. , A Pancancer Analysis of the Clinical and Genomic Characteristics of Multiple Primary Cancers, Scientific Reports. (2024) 14, no. 1, 10.1038/s41598-024-52659-3, 38287125. PMC10825147 38287125

[5] Salhab R. Q. , Ghazaleh Z. I. , Barbarawi W. , Salah-Aldin R. , Hour H. , Sweity R. , and Bakri I. A. , An Unusual Occurrence of Multiple Primary Malignant Neoplasms: A Case Report and Narrative Review, Frontiers in Oncology. (2024) 14, e1381532, 10.3389/fonc.2024.1381532. PMC11288870 39087028

[6] Borja N. A. , Silva-Smith R. , Calfa C. , Sussman D. A. , and Tekin M. , Triple Primary Cancers: An Analysis of Genetic and Environmental Factors, Cancer Prevention Research. (2024) 17, no. 5, 209–215, 10.1158/1940-6207.CAPR-23-0395. 38361103

[7] Zawar A. , Manoj G. , Nair P. P. , Deshpande P. , Suravajhala R. , and Suravajhala P. , Variants of Uncertain Significance: At the Crux of Diagnostic Odyssey, Gene. (2025) 962, 149587, 10.1016/j.gene.2025.149587. 40404072

